# Hearing Aids and the Brain

**DOI:** 10.1155/2014/518967

**Published:** 2014-09-03

**Authors:** K. L. Tremblay, S. Scollie, H. B. Abrams, J. R. Sullivan, C. M. McMahon

**Affiliations:** ^1^Department of Speech and Hearing Sciences, University of Washington, Seattle, WA 98105, USA; ^2^University of Western Ontario, London, ON, Canada N6A 3K7; ^3^Starkey Hearing Technologies, Eden Prairie, MN 55344, USA; ^4^Centre for Language Sciences, Australian Hearing Hub, 16 University Dve, Macquarie University, North Ryde, NSW 2109, Australia

At the heart of most rehabilitation programs for people with hearing loss is the use of amplification. The purpose of hearing aid amplification is to improve a person's access to sound. Depending on the degree and configuration of the individual's hearing loss, the hearing aid is tasked with increasing sound levels at different frequency regions to ensure that incoming speech frequencies are reaching the ear at sufficient levels to compensate for the individual's hearing loss. However, a perceptual event is dependent not only on the audibility of the signal at the level of the ear, but also on how that sound is biologically coded, integrated, and used. As described by Tremblay and Miller in this special issue, this complex ear-brain neural network system starts with sound leaving the hearing aid. At this stage the acoustics of the amplified signal has been altered by the hearing aid. It is this modified signal that is being encoded at subsequent stages of processing: the ear, brainstem, midbrain, and the cortex. The integrity of the signal, and the biological codes, are therefore assumed to contribute to the resultant perceptual event and it is for this reason that the brain can be considered an essential component to rehabilitation. Yet, little is known about how the brain processes amplified sound or how it contributes to perception and the successful use of hearing aid amplification (see Figure 1).

The intent of this IJO special edition is to integrate neuroscience and clinical practice to advance hearing health care. Such lines of inquiry can spawn new insight into stimulation-related brain plasticity that might, in turn, help explain why some individuals (but not others) report increased speech understanding while wearing their devices. From a clinical perspective, there is interest in determining if measures of brain activity might be of use to the clinician, during hearing aid selection and fitting, as well as to the engineers who are designing the instruments. However, to move forward, there are unresolved issues that can appear conflicting to the clinician and/or scientist who wish to embark in new research directions or clinical services. For this reason, a call for papers on the topic of Hearing Aids and the Brain was made in an effort to define converging evidence. What resulted is a collection of papers from different laboratories that identify caveats and concerns, as well as potential opportunities.

The collection of papers addresses two main questions: (1) Is it possible to use brain measures to determine a person's neural detection of amplified sound? (2) Can brain measures provide information about how the brain is making use of this amplified sound? Before we summarize the answers to the questions, it's important to review the framework for including objective measures to examine the neural representation of amplified sound.

When a person is fitted with a hearing aid, there are two categories of test procedures involved in the process: (i) behavioral measures, which require active participation by the patient (e.g., pure-tone threshold estimation, speech testing, and self-report questionnaires), and (ii) objective measures, where subjective responses are not required (e.g., probe microphone electroacoustics and unaided electrophysiology). Here we expand the use of electrophysiology to determine if brain measures can be used as an objective measure to estimate aided thresholds and/or quantify hearing aid transduced signals in the brain for the purpose of guiding device fitting and/or to assess suprathreshold representation of amplified auditory signals in the brain to estimate perceptual performance and/or the related cognitive resources involved. This expanded use of objective measures is relevant to patients of all ages but is particularly germane to the pediatric population where the use of behavioral tools is limited. As described by L. M. Jenstad et al. in this issue, behavioral threshold information is not usually available before age 6 months (and often later), speech testing is unavailable, and subjective questionnaires are limited to caregiver observation of behaviors. Thus, there is greater reliance on objective procedures to measure the effects of amplification beyond the tympanic membrane in infants and young children. One such measure is the use of auditory evoked potentials (AEPs).

The use of AEPs to aid in clinical assessment is not new. Click-evoked auditory brainstem responses were tried long ago to estimate unaided and aided thresholds in infants and young children. However they proved to be unsuccessful because the short duration signal (click, tone-pip) interacted with the hearing aid circuitry in a way that introduced ringing and other artifacts [1]. In this special issue, S. Anderson and N. Kraus reintroduce the concept of using complex speech evoked ABRs (also called the frequency following response (FFR)) and provide a case study to show that it is possible to record FFRs while a person is wearing a hearing aid. But FFR and speech evoked ABR research is still in its infancy. It will become necessary to define the interactions that take place between the instrument and brainstem responses, especially in people with hearing loss and who wear hearing aid devices to determine if some of the obstacles encountered when recording cortical evoked potentials (CAEPs) also apply to the FFR.

There is much literature exploring the role of CAEPs in assessing people with hearing loss, but the inclusion of people with hearing loss who wear hearing aids is still quite sparse. In this special issue, investigators from different laboratories describe some of caveats and concerns when measuring evoked CAEPs in combination with hearing aid amplification. L. M. Jenstad et al. showed that CAEPs (P1-N1-P2) do not reliably reflect hearing aid gain, even when different types of hearing aids (analog and digital) and their parameters (e.g., gain, frequency response) are manipulated. As shown in Figure 2, N1 latency (and N1-P2 amplitudes) is not significantly affected even when the gain of the input signal is increased by 20 dB or 40 dB. This is true even when different hearing aid processing types are involved. These results reinforce those previously published by Billings et al. [2, 3] over the past few years, as well as those presented in this special issue. Billings et al. demonstrate continuing evidence that the signal-to-noise ratio (SNR) produced by the hearing aid and entering the brain influences aided cortical potentials (P1-N1-P2) in a way that obscures the biological representation of hearing aid gain. These results, and those described earlier by Billings et al., provide converging evidence that time-locked cortical activity does not always reflect the expected (and electroacoustically verified) gain of hearing aids, and that different hearing aids with similar electroacoustic parameters (i.e., gain) may result in substantially different evoked results. The use of CAEPs as an accurate proxy of hearing aid gain therefore remains problematic. At issue, in addition to simple gain, is the influence of hearing aid signal processing features such as channel-specific compression time constants, noise reduction algorithms, and adaptive directionality on cortical and subcortical evoked potentials.

L. M. Jenstad et al. describe how these issues impact the clinician. It would be possible to appropriately fit a hearing aid (for degree and configuration of the hearing loss) in a client but show no evoked brain response or no improvement from the unaided cortical response, even though, behaviorally, the client shows clear perceptual improvements. In another manuscript, D. Glista et al. also show instances when cortical P1-N1-P2 responses are absent in normal hearing children. Even when cortical evoked potentials are present, their peak morphology may or may not reflect defining characteristics of the brain response. Instead they could reflect aspects of signal processing influenced by the hearing aid.

We know very little about this last point, and the research published in this issue attests to this. L. M. Jenstad et al. published examples where differences in the type/brand of hearing aid resulted in substantial differences in cortical activity, even though standard electroacoustic measures indicated no major differences among the hearing aids. In contrast, D. Glista et al. presented a few pilot cases where altering frequency compression hearing aid parameters not only improved audibility of a 4 kHz tone burst but also improved detection of cortical evoked responses. So, it appears that the presence of P1-N1-P2 in response to an amplified signal indicates the neural detection of a sound at the level of the cortex, but the morphology of the response (latency and amplitude) may say more about the signal processed acoustics of the amplified sound than the status of the neural mechanisms generating the response. A dilemma ensues when the CAEP is absent. The absence of a brainstem or cortical response, or the absence of change in the evoked brain activity, may result from a number of variables not yet fully understood and therefore complicating the use of AEPs as a sensitive and specific metric of hearing loss and abnormal brain processing. To summarize then, when examining the effect of hearing aid amplification on brain maturation or plasticity, the presence or absence of change over time might simply reflect changes in signal alterations introduced by the hearing aid within a single recording or between sessions. In all cases, changes to the hearing aid prescription can be presumed to contribute to changes in the evoked neural responses. The same can likely be said for cochlear implants too. For any scientist or clinician to overlook the contribution of the hearing aid-transduced signal to stimulation-related patterns in the brain is to overlook a core essential variable.

While the previously expressed concerns highlight the cautionary aspects of combining brain measures and hearing aid amplification, the contribution of device-related changes to evoked brain activity could also be viewed as an opportunity to study the utility of specific hearing aid algorithms in a way that could be used as an outcome measure. This opportunity assumes two things: (1) it is possible to reliably characterize signal processing schemes specific to each device in a way that contributes information to the hearing aid fitting process in an efficient and informative way, and (2) that clinicians (who are reluctant to use existing technology such as probe microphone technology) would be accepting of adding an additional measure to their protocol.

However, as this direction of ear-brain testing continues, future research is needed to explore appropriate test stimuli and presentation paradigms when hearing aids are included in research. As described by D. Glista et al, we need a better understanding of the testing conditions and stimuli that yield the most valid measures electrophysiologically in relationship to behavioral measures. Traditional presentations of speech stimuli, with tones or speech syllables being presented in isolation interleaved with silent periods so the necessary average brain responses which can be obtained, might not be a comparable auditory experience to the running speech that is usually delivered to the brain by the hearing aid. For example, in this issue, V. Easwar et al. evaluated the output levels of 10 different hearing aids for phonemes in isolation and in running speech to determine the effects of processing strategies on the output level of each. Their results show remarkable differences in sound level and hearing aid activation, depending on the method of stimulus presentation. This means that different conclusions could be drawn for the same person, depending on the way in which the stimuli interact with the hearing aid. A more optimistic spin on this finding could be that it might be possible to use cortical activity to assess the effects of hearing aid fine-tuning (e.g., changes to hearing aid gain and frequency shaping), or other aspects of hearing aid signal processing, such as frequency lowering, as illustrated by some participants reported by D. Glista et al. However, validation of aided findings in larger groups of participants with hearing loss including infants and in young children would be needed to establish valid clinical interpretations both for present and absent aided CAEPs. Particularly for assessing the outcome of an infant's hearing aid fitting, appropriate interpretation of an absent CAEP is critical, so that appropriate communication of results to caregivers can take place.

Another important point is that there might be optimal montages for recording brainstem and cortical activity that have not yet been realized. Even though most published data to date show that it is possible to record brainstem and cortical responses using only a small number of electrodes, this need not mean that the routine recording montage typically used in clinical settings is the most sensitive and specific recording approach for research purposes. A recent example of what information can be lost when using a standard vertex recording of the P1-N1-P2 complex in adults can be found in Tremblay et al.[5]. They showed how a significant amount of information about sound processing and relevance to auditory rehabilitation is lost when electrodes over the temporal lobes are not analyzed. This point is especially important when testing the pediatric population. When testing infants and children, recording montages will be influenced by the changing distribution of evoked activity generated by the maturing brain.

Another potential area of research is to examine the interaction between onset and change responses evoked by the auditory cortex. This information provides an objective quantification of the relationship between the onset of the processed signal versus changes within a between processed sounds. The P1-N1-P2 cortical response is appropriate for this purpose because it is sensitive to the onset of sound as well as to acoustic changes within an ongoing sound [4, 5]. When recorded in response to acoustic changes, the same P1-N1-P2 complex is described as an acoustic change complex (ACC). These differences in the CAEPs may have implications for hearing aid-brain interactions. To date little is known about the relationship between hearing aid signal processing in response to the onset of sound and the dynamic changes in hearing aid circuitry that are triggered by the dynamic nature of the speech signal. Rapid and slow dynamic changes in the incoming acoustic signal activate the hearing aid circuitry in different ways that likely influence evoked brain activity measured from the scalp. For this reason, the literature base that is developing around the P1-N1-P2 response may not apply to the acoustic influences contributing to the ACC. Taking this point even further, EEG responses that are not time locked and outside the traditional practice of audiology may prove to be of value to the selection, fitting, and design of hearing aids. The P1-N1-P2 and ACC are being studied because they are already familiar to the audiology community and could be implemented into clinical practice. But there are many ways in which EEG activity can be measured that are not discussed here.

For example, brain measures could guide manufacturer designs. Knowing how the auditory system works and how it responds to gain, noise reduction, and/or compression circuitry could influence future generations of biologically motivated changes in hearing aid design. Biological codes have been harnessed to drive the motion of an artificial limb/prosthesis, it might therefore be possible one day to design a hearing prosthesis that includes neuromachine interface systems driven by a person's listening effort or attention. At this stage, however, finding ways to quantify a person's listening effort is still being defined. For example, demonstrating the benefits of digital noise reduction algorithms, as implemented in hearing aids, on improved speech recognition in noise has been elusive. Anecdotal reports suggest that such algorithms may provide nonspeech perception benefits such as improved ease of listening. Therefore physiologic measures of stress and effort are being explored to determine if they could provide an objective measure of nonspeech benefits. One such example is pupillometry. In this special issue, T. Koelewijn and colleagues were able to demonstrate a difference in the amount of pupil dilation among normal hearing participants listening to speech masked by fluctuating noise versus a single talker. The authors posit that the degree of pupil dilation may reflect the additional cognitive load resulting from the more difficult listening task and suggest that pupillometry may offer a viable objective measure of the benefits associated with specific hearing aid signal processing features such as digital noise reduction.

However, as illustrated by K. L. Tremblay and C. W. Miller, the successful use of hearing aids to improve human communication will ultimately depend on more than just brain measures. Many factors can contribute to aided speech understanding in noisy environments, including device centered (e.g., directional microphones, signal processing, and gain settings) and patient centered variables (e.g., age, attention, motivation, biology, personality, and lifestyle). Research aimed at exploring one variable in isolation (e.g., neural mechanisms underlying sound detection and/or discrimination) is likely to fall short when trying to optimize the many interactive stages involved in human communication.

To summarize and conclude, the collection of articles that appear in this special issue of IJO show that (1) it is possible to use brain measures to determine a person's neural detection of amplified sound and (2) brain measures can provide information about the use of this amplified signal sound. The use of brain measures to quantify and model neural mechanism associated with the perception of amplified sound is complex and sometimes counter-intuitive. For this reason, it is important to remind clinicians and neuroscientists that the interpretation of aided evoked neural activity cannot simply be based on prior published data acquired from normal hearing or unaided participants.

*K. L. Tremblay* *K. L. Tremblay* *S. Scollie* *S. Scollie* *H. B. Abrams* *H. B. Abrams* *J. R. Sullivan* *J. R. Sullivan* *C. M. McMahon* *C. M. McMahon*

## Figures and Tables

**Figure 1:**
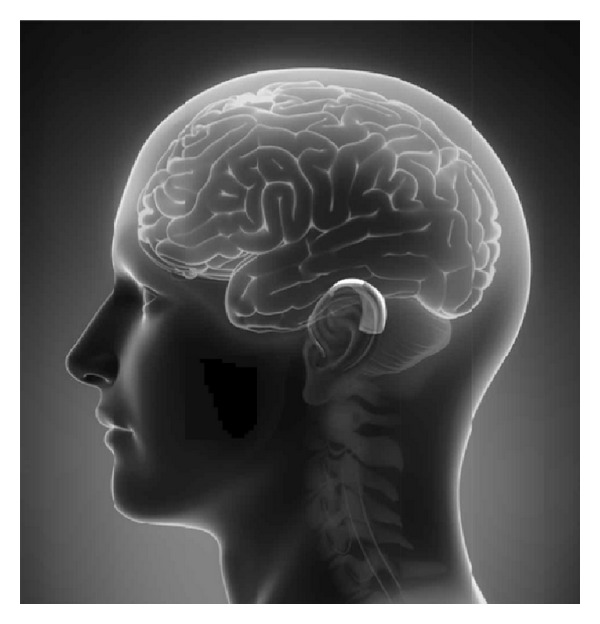


**Figure 2:**
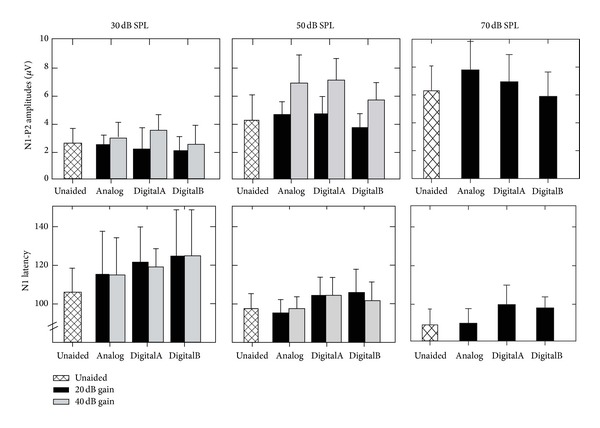
Mean and SD amplitude and latency data for unaided and aided conditions (Analog, DigitalA, and DigitalB) with two gain settings (20 and 40 dB) at three input levels (30, 50, and 70 dB SPL). Reprinted from L. M. Jenstad et al.
